# Supplementary material for: A Comparison of Three Perfusion Algorithms in Patients at Risk of Delayed Cerebral Ischemia After Subarachnoid Hemorrhage
Source: Diagnostics (Basel). 2025 Sep 3;15(17):2236. doi: 10.3390/diagnostics15172236 (PMC12428053; doi:10.3390/diagnostics15172236)
Supplement: Supplementary file 1 [file diagnostics-15-02236-s001.zip › diagnostics-3756237-supplementary.pdf]

Table S1: Perfusion deficit correlated with final infarct development followed by DCI-related treatment

	Volume		Cercare Threshold	Cercare AI	ISP
**DCI-related treatment**		n (%)	56 (100.0)	56 (100.0)	56 (100.0)
	Hypoperfused	mean (range)	20.7 (0.0-185.5)	52.6 (0.0-321.2)	54.1 (2.1-208.4)
	Core	mean (range)	4.5 (0.0-29.5)	9.6 (0.0-57.9)	14.2 (0.0-142.9)
**No abnormality**		n (%)	18 (32.1)	1 (1.8)	0 (0.0)
	Hypoperfused	mean (range)	0 (0.0)		
		n (%)	19 (33.9)	19 (33.9)	1 (1.8)
	Core	mean (range)	0 (0.0)		
**Underestimated progressive infarct**		n (%)	2 (3.6)	0 (0.0)	0 (0.0)
	Hypoperfused	mean (range)	0 (0.0)		
		n (%)	9 (16.1)	5 (8.9)	3 (5.4)
	Core	mean (range)	0 (0.0)		
**Reversible perfusion deficit**		n (%)	28 (50.0)	36 (64.3)	37 (66.1)
	Hypoperfused	mean (range)	25.5 (0.1-185.5)	49.8 (0.2-321.2)	47.5 (4.0-156.2)
		n (%)	18 (32.1)	18 (32.1)	36 (64.3)
	Core	mean in mL(range)	9.2 (0.1-29.3)	20.9 (0.2-57.9)	11.2 (0.1-40.0)
**Progressive Infarct**		n (%)	12 (21.4)	19 (33.9)	19 (33.9)
	Hypoperfused	mean (range)	36.9 (0.3-121.5)	60.5 (0.2-191.4)	67.0 (2.1-208.4)
		n (%)	10 (17.9)	14 (0.25)	16 (28.6)
	Core	mean (range)	8.8 (0.1-29.5)	11.6 (0.2-55.1)	24.5 (0.3-142.9)
Sensitivity	Hypoperfused		85.7%	100.0%	100.0%
Specificty			39.1%	2.7%	0.0%
PPV			30.0%	34.5%	33.9%
NPV			90.0%	100.0%	/
Sensitivity	Core		52.6%	73.7%	84.2%
Specificty			51.4%	51.4%	2.7%
PPV			35.7%	43.8%	30.8%
NPV			67.9%	79.2%	25.0%

Table S2: Perfusion deficit correlated with final infarct development without DCI-related treatment

	Volume		Cercare Threshold	Cercare AI	ISP
**No DCI-related treatment**		n (%)	51 (100.0)	51 (100.0)	51 (100.0)
	Hypoperfused	mean (range)	8.7 (0.0-95.9)	26.8 (0.0-160.0)	32.8 (0.8-131.3)
	Core	mean (range)	1.2 (0.0-13.3)	4.4 (0.0-62.4)	7.1 (0.0-35.6)
**No abnormality**		n (%)	18 (35.3)	1 (2.0)	0 (0.0)
	Hypoperfused	mean (range)	0 (0.0)		
		n (%)	27 (52.9)	22 (43.1)	3 (5.9)
	Core	mean (range)	0 (0.0)		
**Underestimated progressive infarct**		n (%)	2 (3.9)	0 (0.0)	0 (0.0)
	Hypoperfused	mean (range)	0 (0.0)		
		n (%)	6 (11.8)	2 (3.9)	1 (2.0)
	Core	mean (range)	0 (0.0)		
**Reversible perfusion deficit**		n (%)	17 (33.3)	34 (66.7)	35 (68.6)
	Hypoperfused	mean (range)	4.6 (0.2-23.1)	12.2 (0.1-73.1)	24.0 (0.8-82.6)
		n (%)	8 (15.7)	13 (25.5)	32 (62.7)
	Core	mean in mL(range)	1.8 (0.1-5.4)	4.0 (0.1-36.6)	6.7 (0.1-34.9)
**Progressive Infarct**		n (%)	14 (27.5)	16 (31.4)	16 (31.4)
	Hypoperfused	mean (range)	26.2 (0.3-95.9)	59.4 (3.4-160.0)	51.9 (3.4-131.3)
		n (%)	10 (19.6)	14 (27.5)	15
	Core	mean (range)	4.4 (0.1-13.3)	12.3 (0.1-62.4)	9.9 (0.4-35.6)
Sensitivity	Hypoperfused		87.5%	100.0%	100.0%
Specificty			51.4%	2.9%	0.0%
PPV			45.2%	32.0%	31.4%
NPV			90.0%	100.0%	/
Sensitivity	Core		62.5%	87.5%	93.8%
Specificty			77.1%	62.9%	8.6%
PPV			55.6%	51.9%	31.9%
NPV			81.8%	91.7%	75.0%
